# Treatment Patterns, Disease Management, and Quality of Life in Outpatients with CKD and Decreased eGFR: A Multicenter Prospective Observational Study in Greece

**DOI:** 10.3390/jcm15187244

**Published:** 2026-09-17

**Authors:** Evangelia Ntounousi, Kostantinos Stylianou, Smaragdi Marinaki, Vassilios Liakopoulos, Sofia Lionaki, Ioannis Stefanidis, Stylianos Panagoutsos, Dimitrios Goumenos, Dimitrios Xidakis, Ioannis Griveas, Eleni Chelioti, Ioannis Tzanakis, Dimitris Gourlis, Argyris Andreadellis, Emelina Stambolliu, Dimitriοs Petras

**Affiliations:** 1 Department of Nephrology, School of Medicine, University of Ioannina, 45110 Ioannina, Greece; 2 Department of Nephrology, University Hospital of Heraklion, 71500 Heraklion, Greece; 3 Department of Nephrology, Laiko General Hospital, National and Kapodistrian University, 11527 Athens, Greece; 4 Department of Nephrology, AHEPA Hospital, Aristotle University of Thessaloniki, 54636 Thessaloniki, Greece; 5 Department of Nephrology, Attikon University Hospital, National and Kapodistrian University of Athens, 12462 Athens, Greece; 6 Department of Nephrology, University Hospital of Larissa, 41334 Larissa, Greece; 7 Department of Nephrology, University Hospital of Alexandroupolis, 68100 Alexandroupoli, Greece; 8 Department of Nephrology, University Hospital of Patras, 26504 Patras, Greece; 9 Department of Nephrology, Venizelio General Hospital of Heraklion, 71409 Heraklion, Greece; 10 Nephrology Department, Army Share Fund Hospital of Athens, 417 NIMTS, 11521 Athens, Greece; 11 Department of Nephrology, Tzaneio General Hospital, 18536 Piraeus, Greece; 12 Department of Nephrology, General Hospital of Chania, 73300 Chania, Greece; 13 Medical Affairs Department, AstraZeneca, 15123 Athens, Greece; 14 Nephrology Department, Hippokration General Hospital, 11527 Athens, Greece

**Keywords:** chronic kidney disease, disease management, observational prospective study, RASi, SGLT2i, statins, treatment patterns, quality of life

## Abstract

**Background/Objectives**: Chronic kidney disease (CKD) is a major cause of morbidity and mortality. Evidence on routine management and patient-reported outcomes in Greece is limited. The SPIRIT study assessed real-world treatment patterns, disease management, and quality of life (QoL) among CKD patients in Greece. **Methods**: This was a prospective, multicenter, observational study in Outpatient Nephrology Clinics of fifteen National Health System (NHS) Hospitals in Greece enrolling adults with estimated glomerular filtration rate (eGFR) calculated using the chronic kidney disease epidemiology collaboration (CKD-EPI) equation (CKD-EPI eGFR) between 15 and <60 mL/min/1.73 m^2^, with a 12-month follow-up. **Results**: A total of 305 CKD patients participated in the study (40.0% classified as stage G3a, 39.0% G3b, and 21.0% G4), with a mean age of 69.3 years. Antihypertensives were administered to 84.3%, 84.1%, and 85.1% of patients at baseline, 6, and 12 months, respectively, mostly calcium channel blockers, while lipid-lowering agents were administered to 63.3%, 64%, and 63.2% at baseline, 6, and 12 months, respectively, predominantly statins (95.3%, 95.9%, and 96.5% of lipid-lowering users). Interestingly, renin-angiotensin-aldosterone system (RAAS) inhibitors were administered to a total of 160 patients, 91.9% of whom had hypertension, while sodium-glucose cotransporter 2 (SGLT2) inhibitors were administered to 45 patients, with 66.7% of them suffering from CKD and type 2 diabetes. SGLT2i use increased from 15.3% at baseline to 35.4% at 12 months. Mean CKD-EPI eGFR remained stable (mean 40.1, 41.3, and 40.7 mL/min/1.73 m^2^), while median urine albumin-to-creatinine ratio (UACR) increased from 145.2 mg/g at baseline to 253.4 mg/g at 6 months and 246.6 mg/g at 12 months. Statistically significant improvements (*p* < 0.05) were observed between 6 and 12 months in specific domains of the Kidney Disease QoL-Short Form (KDQOL-SF) v1.3. **Conclusions**: The study provides real-world evidence on stage-related treatment patterns, routine management, and QoL in CKD outpatients in Greece.

## 1. Introduction

Chronic kidney disease (CKD) represents a major public health issue, affecting 8–16% [1] of the adult population worldwide, corresponding to over 800 million individuals across all stages [2]. In Greece, CKD is considered as one of the main causes of morbidity and mortality [2] and is responsible for one of the highest rates of kidney failure incidence among developed countries [3]. The national burden is considerable, with more than one million individuals estimated to be affected [4].

Type 2 diabetes mellitus (T2DM) is considered to be the primary risk factor for the development of CKD, increasing the risk of kidney dysfunction more than twofold [5], with diabetic nephropathy representing an important cause of progressive renal dysfunction and end-stage renal disease [6]. Importantly, the relationship between kidney disease and diabetes mellitus is bidirectional. More specifically, chronic hyperglycemia promotes glomerular injury through mechanisms involving inflammation and oxidative stress, thereby contributing to the onset and progression of CKD [7]. In turn, CKD can aggravate insulin resistance and disrupt glucose homeostasis, partly as a result of impaired renal insulin clearance and reduced renal gluconeogenesis, which may adversely affect glycemic control and facilitate the progression of diabetes-related complications [7]. Moreover, hypertension, obesity, and dyslipidemia are prevalent factors that contribute to the initiation and progression of the CKD [8]. CKD patients with T2DM present with a higher mortality rate compared to the general population, while the majority of deaths occur due to atherosclerotic cardiovascular events before progression to kidney failure [9].

Beyond its clinical and physiological consequences, CKD can substantially impair patients’ overall quality of life. As kidney disease progresses, symptoms such as weakness, fatigue, reduced exercise capacity, and cognitive impairment, along with complications such as anemia, fluid and electrolyte disturbances, and cardiovascular comorbidities, may further compromise physical and psychosocial well-being [10]. Factors such as older age, female gender, unemployment, lower education, smoking, and multiple comorbidities are associated with reduced quality of life in CKD patients [11].

Current Kidney Disease: Improving Global Outcomes (KDIGO) guidelines for the management of CKD in diabetic and non-diabetic patients recommend as first line pharmacological treatment the use of renin-angiotensin-aldosterone system inhibitors (RAASi) and early initiation of sodium-glucose cotransporter 2 inhibitors (SGLT2i) [12,13]. Nevertheless, the use of RAAS inhibitors and mineralocorticoid receptor antagonists (MRAs) in everyday practice can be impacted by electrolyte imbalances, especially hyperkalemia, preventing the implementation of ideal treatment patterns [14,15,16]. Lifestyle changes, management of dyslipidemia, and customized care of CKD complications such as anemia, mineral bone dysregulations, and metabolic acidosis represent additional therapeutic measures [8].

With no available national registry for non-dialysis CKD patients in Greece, real-world evidence in the management of this patients group remains limited. To address this issue, we conducted a multicenter, observational, prospective study (SPIRIT study) in order to evaluate treatment patterns, disease management, and quality of life in CKD patients with decreased eGFR who attended hospital-based Nephrology Outpatient Clinics (OCs) for the first time. More specifically, the primary objective of the study was to record prospectively treatment patterns and disease management, including all pharmacological and non-pharmacological interventions, following a CKD diagnosis. The secondary objectives included assessment of health-related quality of life (HRQoL) using two questionnaires, the Kidney Disease Quality of Life-Short Form (KDQOL-SF) v1.3 and the 5-level European Questionnaire (EQ)-5D version (EQ-5D-5L) [index and visual analogue scale (VAS)] at 6 and 12 months, and evaluation of changes in kidney parameters (eGFR by CKD-EPI and MDRD equations, as well as urine albumin-to-creatinine ratio [UACR]/urine albumin) from baseline to 6 and 12 months.

Herein, we summarize SPIRIT study findings and provide insights into current disease management in routine CKD clinical practice in Greece.

## 2. Materials and Methods

### 2.1. Study Design

The SPIRIT study was a multicenter, prospective, national, observational study aiming to record routine clinical practice and treatment patterns in CKD patients with reduced eGFR of 15 to <60 mL/min/1.73 m^2^, presented for the first time to National Health System (NHS) Hospital-based Nephrology OCs in Greece. Patients either booked their appointment on their own initiative or were referred to nephrologists by other medical specialties. The study was conducted over three visits (baseline, 6, and 12 months), in compliance with the Declaration of Helsinki, ICH-GCP, and Good Pharmacoepidemiology Practice (GPP); all participants provided written informed consent and were notified about their withdrawal rights. The study is registered in the Hellenic Registry of Non-Interventional Studies (SFEE RNIS; study code D1843R00342) (https://www.dilon.sfee.gr/studiesp_d.php?meleti_id=D1843R00342 (accessed on 25 June 2026).

### 2.2. Setting, Participants, and Eligibility

Over the course of a nine-month recruitment period, patients who were assessed for the first time at the Nephrology OC of 15 NHS Hospitals were consecutively enrolled. Inclusion criteria were as follows: age ≥ 18 years, CKD-EPI eGFR between 15 and <60 mL/min/1.73 m^2^, and a measurement of UACR or urine albumin within 6 months before study entry or at baseline, regardless of albuminuria level. Exclusion criteria included pregnancy, current SGLT2i therapy, type 1 diabetes, history of kidney transplantation, comorbidity with life expectancy <1 year excluding renal diseases or cardiovascular diseases, and previous nephrological management. To reduce selection bias, investigators enrolled the first twenty consecutive eligible patients per site.

### 2.3. Patient Reported Outcome (PRO) Questionnaires

#### 2.3.1. KDQOL-SF v1.3

The KDQOL-SF™ Version 1.3 was selected to provide a comprehensive assessment of health-related quality of life in patients with CKD by combining the generic SF-36 health survey with kidney disease-specific domains that capture the particular impact of kidney disease on patients’ daily lives [17]. Scoring was conducted in accordance with the official scoring manual. All eight SF-36 subscales and eleven kidney disease–targeted subscales were calculated. Responses were recoded based on their original ordinal scales, and subscale scores were transformed to a 0–100 scale, where higher scores indicate better health status or functioning. For each subscale, scores were computed only when at least 50% of the items were non-missing. The SF-36 domains were summarized into Physical Component Summary (PCS) and Mental Component Summary (MCS) scores. The kidney disease-specific domains included Symptoms/Problems, Effects of Kidney Disease, Burden of Kidney Disease, Cognitive Function, Quality of Social Interaction, Sexual Function, Sleep, Social Support, Work Status, Patient Satisfaction, and Dialysis Staff Encouragement.

#### 2.3.2. EQ-5D-5L

The EQ-5D-5L was selected as a brief, standardized generic measure of health-related quality of life, allowing assessment of overall health status across different health conditions and facilitating comparison of health outcomes across patient populations [18]. In this study, EQ-5D-5L scores were derived from participant responses to the five dimensions of the descriptive system: mobility, self-care, usual activities, pain/discomfort, and anxiety/depression. Each dimension was rated on a 5-level severity scale (1 = no problems to 5 = extreme problems), generating a 5-digit health state profile (e.g., 11223) for each participant at each visit. These health state profiles were converted into index values (utility scores) using the validated EQ-5D-5L valuation algorithm [18]. The resulting index value reflects the relative health-related quality of life associated with each health state, with values typically ranging from below 0 (health states valued as worse than death) to 1 (full health), depending on the applicable value set. Participants also rated their overall health using the EQ-VAS, a vertical visual analogue scale ranging from 0 (worst imaginable health) to 100 (best imaginable health), with higher scores indicating better perceived health. Both the index values and EQ-VAS scores were calculated according to standard scoring guidance [18].

### 2.4. Data Source and Collection Procedures

After a documented feasibility process, 15 nephrology departments based in NHS Hospitals representatively of national nephrology clinical practice were selected. At recruitment, patients’ medical history along with comorbidities were recorded. Study’s clinical and laboratory data were collected during the pre-specified visits, at baseline, at 6 months, and at 12 months. During each visit, pharmacological treatments for each patient were recorded as well. Patients completed the printed PRO questionnaires (EQ-5D-5L and KDQOL-SF v1.3) at each visit. To capture real-world management in a consistent way across all study sites, all data were recorded in an electronic case report form (eCRF).

### 2.5. Statistical Analysis

There was no formal hypothesis testing in this descriptive analysis. Continuous variables were summarized as mean (SD) or median (IQR) per distribution, while categorical variables were presented as counts and percentages. Although not included in percentage denominators, missing counts and percentages are depicted in tables. Data completeness is reported as follows: “Missing *n* (%)” denotes patients with no information on whether the test was performed, and “Not measured *n* (%)” denotes patients for whom the test was intended but not performed; both are expressed as the number (percentage) of patients at the relevant visit. PROs were assessed in agreement with instrument scoring manuals. For the longitudinal PRO analyses, least-squares mean differences (LSMD) were estimated using a mixed-effect model for repeated measures (MMRM), with visit as a fixed effect and patient ID as a random intercept. *p*-values were provided for each comparison, and two-sided *p*-value < 0.05 was considered statistically significant. Post hoc analyses were also conducted to further evaluate UACR changes following SGLT2i administration and to assess the significance of eGFR changes from baseline. Analysis of UACR changes included descriptive and longitudinal inferential components. Longitudinal changes in eGFR, renal function, and albuminuria were analyzed using unadjusted linear mixed-effects models, with time treated as a categorical fixed effect and patient as a random intercept to account for within-subject correlation of repeated measurements. Two-sided *p*-values < 0.05 were considered statistically significant. A designated Contract Research Organization (CRO) performed all analyses, using SAS^®^ version 9.4.

## 3. Results

### 3.1. Patient Disposition and Characteristics at Baseline

**Figure 1:**
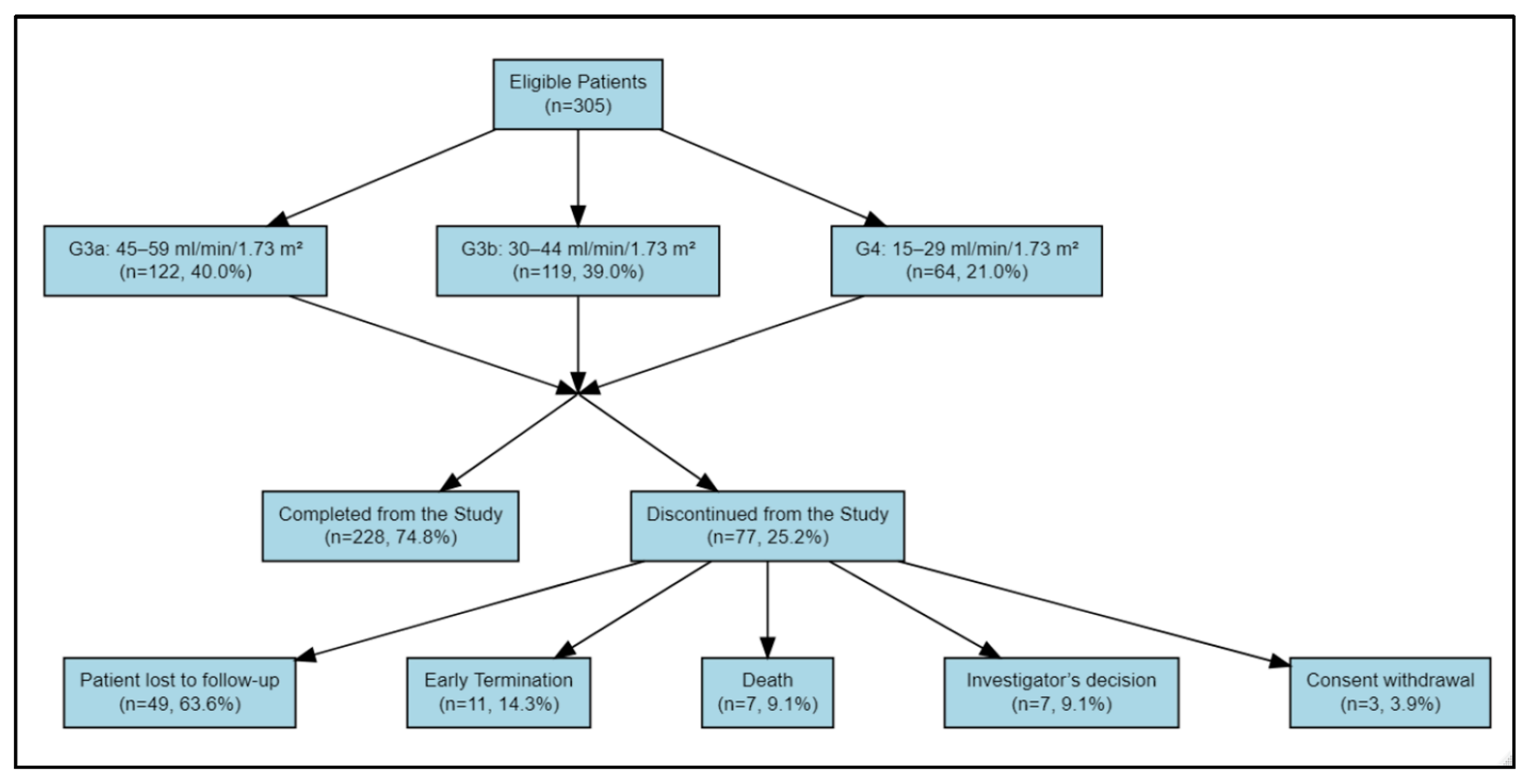
Patients flowchart.

Of the 309 patients that underwent screening, four did not meet the eligibility criteria, leaving 305 patients in the final cohort. In total, 122 (40%) out of 305 patients were classified as CKD stage G3a, 119 (39.0%) as G3b, and 64 (21.0%) as G4, respectively (Figure 1). Baseline demographics, lifestyle habits, and comorbidities are summarized in Table 1. Regarding comorbidities, as reported by the investigators, arterial hypertension (HT) was the most prevalent one (73.4%), with slightly higher rates in stage G4 (78.1%) compared to G3a (70.5%) and G3b (73.9%), followed by uncontrolled dyslipidemia (34.4%) and T2DM (29.8%), Table 1.

### 3.2. Disease History and Characteristics

Disease history and characteristics are presented in Table 2. Family history of CKD was reported in 6.6% of participants, while HT (46.2%) and T2DM (29.2%) were the main causes of reduced eGFR, as reported by the investigators (Table 2).

### 3.3. Laboratory Assessments and Physician’s Observations

Laboratory results during the study are summarized in Table 3 and Table S1. Baseline median UACR level in the total cohort of 302 patients was moderately increased at 145.2 mg/g (IQR: 28.9–640.1). Notably, in patients with available UACR levels during follow-up, median UACR levels further increased at 253.4 mg/g (IQR: 48.4–649.2) in 142 patients and 246.6 mg/g (IQR: 43.5–654.2) in 156 patients, at months 6 and 12, respectively (Table 3). Longitudinal changes observed in albuminuria-related markers were estimated by using LMM and showed that these changes were not statistically significant (Table S2). A post hoc analysis was also performed to further evaluate UACR and its components in patients receiving SGLT2i (n = 45, 15 non-diabetic, 30 diabetic). In non-diabetic patients, median UACR decreased from 70.3 mg/g (IQR: 51.3–466.4) at baseline to 47.4 mg/g (IQR: 26.5–553.5) at the end of the study. In diabetic patients, median UACR values remained relatively stable throughout the study: 36.6 mg/g (IQR: 15.9–93.0) at baseline, 35.6 mg/g (IQR: 14.9–84.0) at 6 months, and 33.2 mg/g (IQR: 13.6–134.3) at 12 months. Similarly, in the combined SGLT2i cohort, median UACR remained relatively stable, with minor fluctuations over time (from 45.1 mg/g IQR: 20.3–180.5] at baseline to 52.4 mg/g [IQR: 22.1–240.6] at 6 months, and 40.8 mg/g [IQR: 18.9–210.7] at 12 months) (Table S3).

Evaluation of renal function over time using both the CKD-EPI and MDRD formulas, based on descriptive comparisons at each timepoint, indicated minimal mean changes in eGFR from baseline to follow-up visits, which did not reach statistical significance (Table 3). Specifically, at 6 months, the mean change in CKD-EPI eGFR was +1.0 mL/min/1.73 m^2^ (SD: 9.1), and in MDRD eGFR, it was +0.9 mL/min/1.73 m^2^ (SD: 8.1). At 12 months, CKD-EPI showed a slight decline with a mean change of −0.5 mL/min/1.73 m^2^ (SD: 9.9), while MDRD eGFR had a similar small reduction of −0.4 mL/min/1.73 m^2^ (SD: 8.7) (Table 3). To further evaluate possible renal function changes over time, a post hoc analysis using LMM was performed. This model-based approach, which accounts for repeated measurements and incorporates all available data, demonstrated a statistically significant increase in CKD-EPI eGFR at 6 months compared with baseline (estimated mean difference +1.5 mL/min/1.73 m^2^; 95% CI: 0.3–2.7, *p* = 0.01), whereas no significant difference was detected at 12 months (*p* = 0.8). Similarly, MDRD eGFR statistically significantly increased at 6 months (+1.4 mL/min/1.73 m^2^; 95% CI: 0.3–2.5, *p* = 0.01), with no significant difference at 12 months (*p* = 0.8) (Table S2).

Most laboratory markers demonstrated comparable values across study visits, with the exception of parathyroid hormone (PTH), which showed greater variability and higher median values at 6, compared to baseline (Table S1).

Patients during follow-up (FU) were referred to other medical specialties, including cardiological, neurological, ophthalmological assessments, and imaging tests, though the frequency varied across visits. Specifically, electrocardiograms (ECGs) were performed on 52 patients (17.0%) at baseline and 25 (11.0%) at 12 months. Clinically significant abnormalities were observed in five and in one patient, respectively, including bradycardia and atrial fibrillation. Heart ultrasound and triplex was conducted in 38 (12.5%) cases at baseline and 26 (11.4%) at 12 months, with clinically significant abnormalities observed in 17 and 4 patients, respectively. Further on, neurological examinations were conducted in 20 patients (6.6%) at baseline and 8 (3.5%) at 12 months, while ophthalmological assessment was performed in 27 patients (8.9%) at baseline and 4 (1.8%) at 12 months, identifying clinically significant abnormalities in 29.6% and 50.0% of them, respectively, including diabetic retinopathy and moderate hypertensive retinopathy. Finally, kidney imaging was conducted in 94 patients (30.8%) at baseline, 22 (8.3%) at 6 months, and 26 (11.4%) at 12 months, with clinically significant findings in 42.6%, 22.7%, and 3.8% of examinations respectively.

### 3.4. Treatment Patterns Across Study Visits and CKD Stages

**Figure 2:**
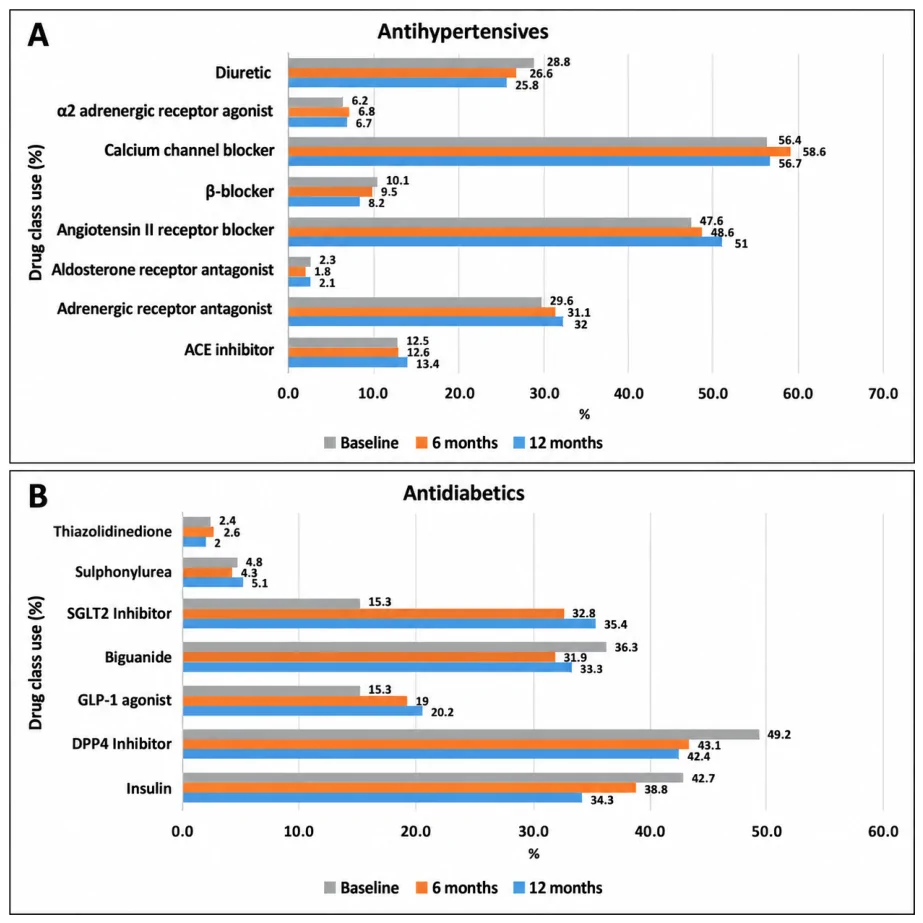
Drug subcategories administered across study visits for (**A**) antihypertensives and (**B**) antidiabetics. Percentages of patients receiving each treatment per study visit are presented.

Overall treatment patterns were consistent across visits, with the number of antihypertensive drugs rising as the most frequently administered type of therapy (used in 84.3% of patients at baseline, 84.1% at 6 months, and 85.1% at 12 months), followed by lipid-lowering agents (administered in 63.3% of patients at baseline, 64% at 6 months, and 63.2% at 12 months). Treatments’ distribution per CKD stage during the study is presented in Table S4. Among antihypertensive drugs, calcium channel blockers (CCB) and angiotensin II receptor blockers (ARB) were the most commonly used across study visits (Figure 2A), while statins represented the most common lipid-lowering agent (Figure S1). Antidiabetics were also frequently used throughout the study (administered to 40.7% of patients at baseline, 43.9% at 6 months, and 43.4% at 12 months), with the most frequently administered antidiabetic medications among patients with T2D being DPP4 inhibitors, followed by insulin (Figure 2B). It should be mentioned that both insulin (42.7% at baseline to 34.3% at 12 months) and DPP-4 inhibitor (49.2% at baseline to 42.4% at 12 months) use decreased throughout the study. On the contrary, the use of SGLT2 inhibitors presented the most notable change, increasing from 15.3% at baseline to 32.8% at 6 months and 35.4% at 12 months (Figure 2B). Other drug subcategories’ distribution is depicted in Figure S1.

**Figure 3:**
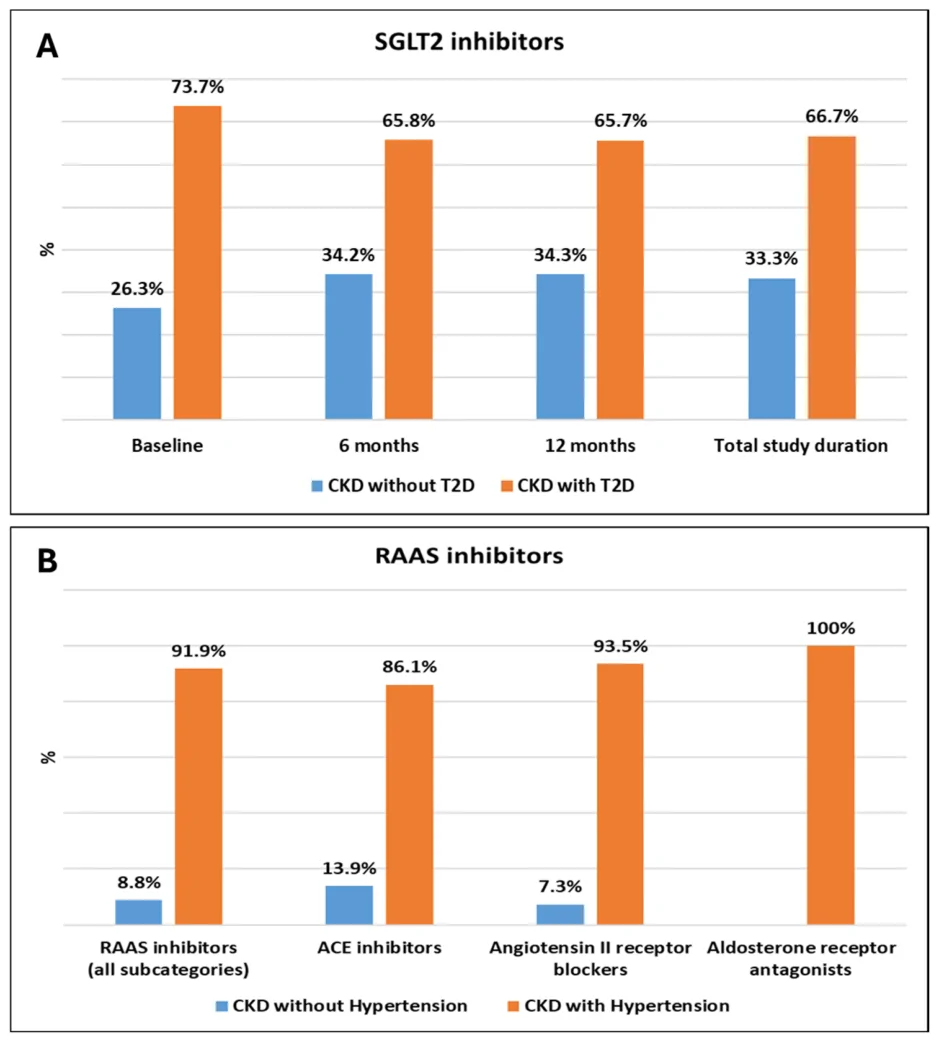
Disease management in CKD patients with diabetes and in CKD patients with hypertension. (**A**): Use of SGLT2 inhibitors among CKD patients with vs. without type 2 diabetes (T2D). (**B**): Use of RAAS inhibitors and respective subcategories among CKD patients with vs. without hypertension.

Overall treatment and pharmacological agents’ categories distribution across CKD stages demonstrated a CKD severity-dependent variation (Figure S2). The observed use of ARBs at baseline reached its highest level in the G3a cohort (66.7%) and decreased in G4 (25.4%), whereas diuretics use increased as the disease progressed (28.1% in G3a versus 39.6% in G4). The use of biguanide decreased with stage progression (e.g., for 12 months: 34.8% in G3a vs. 10.0% in G4), while insulin and DPP-4 inhibitors were generally more common in advanced CKD. The number of patients receiving SGLT2 inhibitors at 12 months was higher in the G3a (39.1%) than in the G4 (25.0%) cohort. Among patients receiving SGLT2 inhibitors (n = 45), 66.7% suffered from both CKD and T2DM (Figure 3A). In the same context, among patients receiving RAAS inhibitors (RAASi) (n = 160), 91.9% were treated for CKD with coexisting hypertension (Figure 3B).

### 3.5. Health-Related Patient-Reported Outcomes

The KDQOL-SF v1.3 and EQ-5D-5L (VAS scale) questionnaires were used to assess HRQoL, with higher scores indicating better perceived health status and functioning. HRQoL, as assessed through the KDQOL-SF v1.3 questionnaire, remained relatively stable throughout the study, with minor variations across domains. At Visit 1, mean scores were generally high across kidney-specific and general domains. Patients reported strong cognitive function (mean: 85.0), minimal perceived effects of kidney disease (85.2), and high social support (85.1), while general health (53.0) and energy/fatigue (60.8) subscales scored comparatively lower. The emotional well-being domain showed moderate results (68.8), and physical and role-based limitations (physical functioning: 65.4; role physical: 63.2) suggested some impairment (Table S5). It should be noted that on the second and third visits, fewer patients filled in the two questionnaires, in particular 256 patients at 6 months and 222 patients at 12 months. By Visit 2, descriptive scores were either stable or slightly improved across most domains, with notable increases in cognitive function (88.5), emotional well-being (72.6), and symptom/problem list (87.4), while physical composite scores remained consistent (SF-12 PCS: 44.6; MCS: 50.1). At Visit 3, scores largely persisted, with minor declines in domains like cognitive function (85.8) and sleep (73.7), though overall health (71.4) and symptom/problem burden (86.6) remained steady. Notably, the social domains (social function: 75.7; quality of social interaction: 78.2) were consistently preserved across visits (Table S5). Moreover, comparative analyses were performed using an MMRM model, allowing inclusion of patients with incomplete questionnaires across visits and accounting for within-patient correlations over time.

According to patients’ responses, at 6 months, a statistically significant reduction (MMRM-based) from baseline was observed in the following domains: cognitive function, emotional well-being, energy/fatigue, symptom/problem list, social function, and mental health composite (SF-12 MCS), indicating a transient worsening in psychosocial and mental aspects of HRQoL (*p* < 0.05 for all). However, by Visit 3 (12 months), most parameters presented similar to baseline levels, with no statistically significant differences from baseline in most domains. Between 6 and 12 months, significant improvements were observed in the effects of kidney disease (*p* = 0.002), cognitive function (*p* = 0.029), and quality of social interaction (*p* = 0.011) (Table S5). EQ-5D-5L outcomes were collected throughout the study to assess participants’ HRQoL from both standardized health utility and subjective perspectives (Table S6). EQ-5D-5L index values remained stable across visits with a median score of 0.9 (IQR: 0.8–1.0). The EQ-VAS scores, which captured patients’ self-rated overall health, showed a slight upward trajectory: from a median of 75.0 at baseline (304 patients) and 6 months (261 patients) to 80.0 at 12 months (226 patients), suggesting a modest improvement in patients’ self-reported general health over time.

## 4. Discussion

The SPIRIT study is the first multicenter, prospective, observational study designed to describe the management of CKD patients in NHS hospital-based OC of Nephrology Departments in Greece. This study provides an overview of patients’ baseline characteristics and comorbidity status at first nephrology evaluation, along with prospective longitudinal assessment of established kidney function parameters, relevant laboratory parameters, administered medications, and patient-reported HRQoL outcomes, over 12 months of routine nephrology care.

Throughout the study period, renal function remained almost stable, with no significant longitudinal changes observed in eGFR levels as calculated by both CKD-EPI and MDRD eGFR equations. In particular, the post hoc analysis revealed a small but statistically significant increase in eGFR at 6 months, which returned to baseline levels at 12 months. This finding aligns with previous evidence suggesting that in CKD stages 3–4 patients undergoing regular nephrology care, eGFR deterioration can be minimal during similar follow-up periods [19,20]. In contrast, median UACR levels were shown to increase from baseline towards study end, although median eGFR level remained stable. It is important to point out the fact that almost half of the baseline UACR measurements lacked follow-up counterparts at 6 and 12 months (302 samples at baseline vs. 142 samples at 6 months and 156 at 12 months), and a comprehensive head-to-head comparison was not feasible, which is among the study limitations. Regarding the rest of the assessed laboratory markers, hemoglobin, serum albumin, glycemic markers, electrolytes, and lipid profile markers showed no substantial change in the total cohort. Overall, the aforementioned findings demonstrate relative stability of renal function over the 12-month follow-up period, although, given the observational design and short follow-up duration, they do not allow definitive attribution of this stability to nephrology care. They nevertheless highlight the potential importance of regular nephrology follow-up in monitoring renal function and progression risk factors in CKD.

Pharmacological treatment in the total cohort remained largely consistent throughout the study period. Therapeutic adjustments following the initiation of nephrological follow-up were specifically aimed at optimizing the management of modifiable risk factors, tailored to the severity of CKD [12,13,21,22]. Antihypertensive agents were the most administered medications, with RAASi being the most commonly used in 64.4% of the total cohort at study end. Among them, ARBs were preferred over ACEis in 51% vs. 13.4% of the patients at 12 months, while CCBs were administered in 56.7% of the cohort. Nevertheless, RAASi use involved only a subset of the overall CKD population, despite the high burden of hypertension and albuminuria, highlighting their relatively limited use in this real-world cohort. This observation may reflect potential real-world barriers to broader implementation even from nephrologists. However, the study was not designed to assess treatment eligibility, contraindications, or the clinical rationale for non-prescription, which should therefore be taken into consideration when interpreting the observed treatment patterns. Indeed, ARBs use was more common in mild to moderate disease stages (G3a, G3b), while diuretics use generally increased as CKD progressed (G4). These patterns may reflect differences in treatment considerations across CKD stages to prevent hyperkalemia and manage higher volume requirements. Aldosterone receptor antagonists’ administration remained limited, possibly due to concerns regarding hyperkalemia risk in advanced CKD stages, although current guidelines support their cautious use in persistent hypertension or albuminuric CKD with close monitoring [12]. Lipid-lowering agents were administered to almost 63% of the total cohort. In particular, the utilization rates of lipid-lowering agents at baseline were 66.4% in stage G3a, 68.1% in G3b, and lower in stage G4 48.4%. Statins emerged as the predominant therapeutic intervention for dyslipidemia management with more than 90% of the population receiving these agents.

Almost one third of the total cohort were T2DM patients. Antidiabetic medication at baseline involved mostly DPP-4 inhibitors (49.2%), insulin (42.7%), and metformin-based regimens (36.3%), in agreement with previously reported data and to some extent with current guidelines mainly aiming for blood glucose control [12,22,23,24,25,26,27]. On the contrary, the percentage of patients receiving SGLT2 inhibitors at baseline was very low, only 15.3% of patients, mostly diabetics, considering the fact that the majority of study patients had indication to receive SGLT2 inhibitors according to prevailing clinical practice. At this point, it should be noted that the initiation of SPIRIT study coincided with the early stages of dapagliflozin reimbursement for non-diabetic CKD indications in Greece. During this transitional period, administration of SGLT2 inhibitors for cardio-kidney protection was limited due to regulatory framework requirements and the medications’ high cost, which may have contributed to its initially limited uptake only among a small percentage of T2DM patients. The following upward trend in SGLT2 inhibitors administration rates over the 12 months, to 35.4%, coupled with a marginal decline in insulin and DPP-4 inhibitor use, reflects a gradual shift toward implementation of guideline-directed medical therapy. Notably, SGLT2 inhibitors during nephrology management were administered to both diabetic and non-diabetic patients with CKD in all stages. This pharmacological therapeutic pattern is in line with the established cardio-kidney beneficial effect of SGLT2 inhibitors in this high-risk population, which originates from large randomized controlled trials (RCTs) and from accumulating real-world evidence, far beyond established glycemic indications [27,28,29].

Moreover, our data showed that in the overall SGLT2i-treated cohort (45 patients), median UACR remained relatively stable over time, with a slight reduction observed at 12 months compared with baseline. A similar pattern was noted in the fifteen non-diabetic patients, in whom UACR decreased from 70.3 mg/g at baseline to 47.4 mg/g by the end of follow-up, whereas UACR median level remained stable in diabetic patients. Although these findings should be interpreted with caution given the relatively small sample size, they might support the beneficial effect of SGLT2 inhibitors on albuminuria in non-diabetic CKD patients. Indeed, as mentioned before, this observation is consistent with previously published RCTs evidence and real-world data, demonstrating that SGLT2i could reduce the risk of CKD progression and are associated with a slower rate of kidney function decline. Moreover, SGLT2 inhibition has been shown to improve cardiovascular and heart failure outcomes across the full spectrum of kidney function and albuminuria levels in patients with CKD, both with and without T2DM [30,31,32,33]. Despite the gradual increase in SGLT2 inhibitors administration following nephrology assessment and monitoring, the overall uptake of SGLT2 inhibitors remained relatively limited within this cohort, highlighting a recorded from previous international observational studies potential gap between evidence-based cardio-renal benefits and their implementation in routine nephrology care [13,29].

Estimated GFR and albuminuria constitute the cornerstones of both the diagnostic and monitoring framework in CKD, and it is well known that their dual assessment provides a robust predictive model for CKD progression and associated cardiovascular risk. In this context and taken together with the observed shifts in pharmacological treatment patterns during SPIRIT study, cohort-level eGFR remained stable for 12 months. Regarding UACR levels, results varied. In the total cohort, median UACR levels appeared to increase and remained elevated until study end. This discrepancy might therefore agree with previous evidence suggesting that albuminuria may worsen even with unchanged eGFR, a pattern that, besides predicting adverse renal outcomes, also highlights the need for a targeted approach to monitoring and treatment in real-world practice [13,29,34]. Nevertheless, we have to consider that this result might be partially explained by the fact that half of the baseline UACR measurements lacked follow-up counterparts at 6 and 12 months, which may limit the interpretation of UACR trends over time and should be taken into consideration when interpreting changes in albuminuria. On the other hand, in the SGLT2i-treated cohort (45 patients), median UACR showed a slight reduction at 12 months compared with baseline. Overall, given the stable eGFR but persistent albuminuria, the SPIRIT study findings support continued focus on albuminuria-targeted management in routine practice [12,35,36,37,38]. This observation corresponds with international requests for a more extensive therapeutic emphasis on albuminuria reduction as an indicator of renal and cardiovascular protection.

Regarding study population characteristics, the mean age at baseline was 69.3 years, consistent with reported international patient populations with stage G3-G4 CKD [39,40]. A stage-dependent gender distribution was identified, with males predominant in G3a/G3b and females in the G4 stage, while BMI was similar across stages, in agreement with previously published Greek data [41]. The above baseline characteristics were accompanied by the expected CKD etiologies and comorbidities. The most prevalent risk factors and possibly primary CKD causes (no biopsy available in all patients) were HT (73.4%) and T2DM (29.8%), and this result is in alignment with previously reported data on major CKD risk factors [42,43,44,45]. Other comorbidities, such as uncontrolled dyslipidemia and hyperuricemia, were frequently reported, highlighting the overall high cardiovascular risk of our population, which has been previously recorded in CKD [46,47].

The SPIRIT study also explored patient-reported measures of HRQoL as an integral part of CKD management, by utilization of EQ-5D-5L and KDQOL-SF questionnaires. Over the 12-month study period, EQ-5D-5L index values remained statistically stable, while EQ-VAS scores showed a small but significant improvement between baseline and 12 months, suggesting that although utility-based health profiles remained stable, participants perceived a modest improvement in their overall health status. Moreover, modest changes in KDQOL-SF v1.3 subscales were recorded, with a temporary decline at 6 months, followed by a recovery to higher levels at 12 months. Overall, these findings are consistent with previously published data, demonstrating relatively stable HRQoL in CKD patients under routine nephrology care, with fluctuations predominantly affecting psychosocial aspects over time [48,49,50,51]. It should be noted that the herein observed stability of HRQoL over the 12-month follow-up might be partially attributed to the relative stability of renal function, as reflected by cohort-level eGFR, as well as other laboratory parameters, such as hemoglobin, a clinically relevant marker of anemia in CKD, during the study period.

The study’s key strengths include its prospective, multicenter design, sufficient sample size, sustained patients’ participation across visits, and comprehensive capture of both clinical and patient-reported parameters. As a non-interventional study, SPIRIT is also subject to limitations typical of real-world observational design, such as the absence of a control group, the lack of repeated samples in a number of the evaluated biomarkers, i.e., albuminuria and the potential for selection, confounding, and information bias. Nevertheless, by collecting data from 15 NHS Nephrology OC nationwide, the study provides an extensive overview of routine CKD management in Greece.

## 5. Conclusions

In conclusion, the SPIRIT study is the first attempt to assess and highlight critical aspects of CKD management in a nephrology outpatient setting in Greece. Study results depict the high burden of hypertension, T2DM, and dyslipidemia, in moderate to severe CKD, hence the need for integrated monitoring of both renal and cardiovascular risk factors. Although SGLT2 inhibitor usage increased in routine clinical practice, reflecting a shift towards adherence to guidelines in terms of kidney protection, broader implementation remains essential. The study’s findings collectively underscore the importance of prompt nephrology referral, timely diagnosis, and systematic management to optimize clinical outcomes in CKD patients in Greece.

## Figures and Tables

**Table 1:** Baseline characteristics and comorbidities stratified by CKD stages.

	G3a *n* = 122 (40.0%)	G3b *n* = 119 (39.0%)	G4 *n* = 64 (21.0%)	Total *n* = 305 (100.0%)
***Demographics***				
**Age (years)**				
N	122	119	64	305
Mean (SD)	66.3 (12.1)	70.9 (11.4)	72.0 (12.6)	69.3 (12.2)
**Gender, *n* (%)**				
Female	21 (17.2)	41 (34.5)	34 (53.1)	96 (31.5)
Male	101 (82.8)	78 (65.5)	30 (46.9)	209 (68.5)
**Race, *n* (%)**				
White/Caucasian	118 (96.7)	117 (98.3)	61 (95.3)	296 (97.0)
Asian	2 (1.6)	-	-	2 (0.7)
Black	1 (0.8)	-	2 (3.1)	3 (1.0)
Other	1 (0.8)	2 (1.7)	1 (1.6)	4 (1.3)
**BMI** ^**[1]**^ **(kg/m^2^)**				
N	122	119	64	305
Median (IQR)	27.9 (25.5, 31.5)	27.9 (25.1, 31.1)	27.1 (25.2, 30.9)	27.7 (25.4, 31.3)
***Lifestyle habits***				
**Exercise status**				
Sedentary	58 (47.5)	55 (46.2)	31 (48.4)	144 (47.2)
Lightly active	30 (24.6)	45 (37.8)	24 (37.5)	99 (32.5)
Moderately active	24 (19.7)	16 (13.4)	8 (12.5)	48 (15.7)
Very active	10 (8.2)	2 (1.7)	1 (1.6)	13 (4.3)
Missing	-	1 (0.8%)	-	1 (0.3%)
**Smoking status**				
Current	21 (17.2)	18 (15.1)	8 (12.5)	47 (15.4)
Never	63 (51.6)	71 (59.7)	39 (60.9)	173 (56.7)
Quit >3 years	25 (20.5)	21 (17.6)	12 (18.8)	58 (19.0)
Quit ≤3 years	11 (9.0)	7 (5.9)	5 (7.8)	23 (7.5)
Missing	2 (1.6%)	2 (1.7%)	-	4 (1.3%)
**Alcohol intake** ^**[2]**^				
<1 unit/day	64 (52.5)	54 (45.4)	27 (42.2)	145 (47.5)
>1 unit/day	50 (41.0)	60 (50.4)	35 (54.7)	145 (47.5)
Never	5 (4.1)	4 (3.4)	1 (1.6)	10 (3.3)
Missing	3 (2.5%)	1 (0.8%)	1 (1.6%)	5 (1.6%)
**Dietary salt restriction**				
Yes	68 (55.7)	65 (54.6)	44 (68.8)	177 (58.0)
Missing	4 (3.3%)	7 (5.9%)	4 (6.3%)	15 (4.9%)
***Comorbidities*** ^**[3]**^***, n (%)***				
Arterial hypertension	86 (70.5)	88 (73.9)	50 (78.1)	224 (73.4)
Type 2 diabetes mellitus	32 (26.2)	44 (37.0)	15 (23.4)	91 (29.8)
Uncontrolled Dyslipidemia	41 (33.6)	46 (38.7)	18 (28.1)	105 (34.4)
Hyperuricemia	19 (15.6)	26 (21.8)	14 (21.9)	59 (19.3)
Coronary artery disease	13 (10.7)	22 (18.5)	8 (12.5)	43 (14.1)
Hypothyroidism	9 (7.4)	14 (11.8)	7 (10.9)	30 (9.8)
Atrial fibrillation	6 (4.9)	7 (5.9)	4 (6.2)	17 (5.6)
COPD	3 (2.5)	5 (4.2)	1 (1.6)	9 (3.0)
Depression	2 (1.6)	5 (4.2)	2 (3.1)	9 (3.0)
Heart failure	1 (0.8)	4 (3.4)	5 (7.8)	10 (3.3)
Missing	6 (9.4)	7 (10.4)	5 (13.2)	18 (10.7)

CKD stages according to eGFR calculation, based on routine plasma creatinine measurement by hospital-based nephrologists. ^**[1]**^ BMI = [weight (kg)/height (cm)^2^] × 10,000. ^**[2]**^ 1 alcohol unit = 1 glass of wine (90 mL) or ½ bottle of beer (250 mL) or 1 measure of spirit. ^**[3]**^ Patients with more than one comorbidity are included in more than one row. BMI = Body Mass Index; IQR = interquartile range.

**Table 2:** Medical history and disease characteristics.

	Total N = 305 (100.0%)
**Time from first report of reduced eGFR to Informed Consent Form (ICF) date (days)**	
N	304
Median (IQR)	140 (22–602.5)
Missing, *n* (%)	1 (0.3%)
**Family History of CKD, *n* (%)**	
Yes	20 (6.6%)
No	285 (93.4%)
Missing, *n* (%)	-
**Possible Causes of reduced eGFR, *n* (%)** ^**[1], [2]**^	
Type 2 diabetes mellitus	89 (29.2%)
Arterial Hypertension	141 (46.2%)
Obesity	15 (4.9%)
Glomerular diseases/Glomerulonephritis	22 (7.2%)
Tubulointerstitial diseases/interstitial nephritis	3 (1%)
Polycystic kidney disease or other inherited kidney diseases	4 (1.3%)
Other cardiovascular disease *n* (%)	15 (4.9%)
Other causes of reduced eGFR, *n* (%)	97 (31.8%)

^**[1]**^ More than one possible cause of reduced eGFR was recorded per patient. ^**[2]**^ Histopathological confirmation was not available for all patients; therefore, the assessment was based on clinical and laboratory findings. CAD = coronary artery disease; IQR = interquartile range.

**Table 3:** Kidney function parameters over time.

	Baseline	6 Months	12 Months
***Renal function***	N = 305	N = 264	N = 228
**Serum creatinine (mg/dL)**			
N	305	256	228
Median (IQR)	1.7 (1.4, 2.1)	1.7 (1.4, 2.1)	1.7 (1.4, 2.2)
**CKD-EPI eGFR (mL/min/1.73 m^2^)**			
N	305	256	228
Mean (SD)	40.1 (11.9)	41.3 (15.3)	40.7 (15.5)
**MDRD eGFR (mL/min/1.73 m^2^)**			
N	305	256	228
Mean (SD)	37.3 (10.6)	38.5 (13.8)	37.8 (13.7)
**Change in CKD-EPI eGFR from baseline**			
N	222	222	222
Mean Change (SD)	—	1.0 (9.1)	−0.5 (9.9)
*p*-value	—	0.119	0.489
**Change in MDRD eGFR from baseline**			
N	222	222	222
Mean Change (SD)	—	0.9 (8.1)	−0.4 (8.7)
*p*-value	—	0.117	0.457
***Albuminuria***	N = 305 ^[1]^	N = 264	N = 228
**Urinary albumin (mg/L)**			
N	305	142	156
Median (IQR)	98.0 (20.0, 436.0)	130.0 (28.0, 405.0)	140.0 (26.0, 471.0)
Missing, *n* (%)	—	122 (46.2%)	72 (31.6%)
**Urinary creatinine (g/dL)**			
N	302	142	156
Median (IQR)	0.07 (0.05, 0.11)	0.06 (0.01, 0.09)	0.08 (0.05, 0.09)
Missing, *n* (%)	3 (1.0%)	122 (46.2%)	72 (31.6%)
**UACR (mg/g)**			
N	302	142	156
Median (IQR)	145.2 (28.9, 640.1)	253.4 (48.4, 649.2)	246.6 (43.5, 654.2)
Missing, *n* (%)	3 (1.0%)	122 (46.2%)	72 (31.6%)

^[1]^ Measured within 6 months from baseline. CKD = chronic kidney disease; CKD-EPI = Chronic Kidney Disease Epidemiology Collaboration (equation for eGFR); eGFR = estimated glomerular filtration rate; IQR = interquartile range; MDRD = Modification of Diet in Renal Disease (equation for eGFR); SD = standard deviation; UACR = urinary albumin-to-creatinine ratio.

## Data Availability

The datasets used and analyzed during the current study are available from the corresponding author on reasonable request.

## Supplementary Materials

The following supporting information can be downloaded at: https://www.mdpi.com/article/10.3390/jcm15187244/s1, Figure S1. Drug subcategories administered across study visits for: A. Antihypertensives, B. Lipid lowering agents, C. Antidiabetics, D. Antiplatelets/Anticoagulants, E. Antibiotics/Antituberculous, F. NSAIDs. Percentages of patients receiving each treatment per study visit are presented. Figure S2. Patterns in overall use of A. Antihypertensives, B. Antidiabetics, C. Lipid Lowering agents, D. Antiplatelets/Anticoagulants, E. Antibiotics/Antituberculous, F. Corticosteroids, and G. NSAIDs, across CKD stages, stratified by study visit. Table S1. Clinical monitoring of other laboratory parameters over time. Table S2. Estimated changes from baseline in renal function and albuminuria over time based on linear mixed-effects models. Table S3. Analysis on UACR change after SGLT2i administration. Table S4. Treatments Distribution During the Study Stratified by CKD Stage. Table S5. KDQOL-SF v1.3 Domain Scores Stratified by Visit and Comparative analysis (MMRM) between Study Visits. Table S6. EQ-5D-5L Metrics during the Study, and Comparative Analysis between Study Vis-its.
