# Supplementary material for: Treatment Patterns, Disease Management, and Quality of Life in Outpatients with CKD and Decreased eGFR: A Multicenter Prospective Observational Study in Greece
Source: J Clin Med. 2026 Sep 17;15(18):7244. doi: 10.3390/jcm15187244 (PMC13607092; doi:10.3390/jcm15187244)
Supplement: Supplementary file 1 [file jcm-15-07244-s001.zip › jcm-4428965-supplementary.pdf]

Article

Treatment Patterns, Disease Management, and Quality of Life in Outpatients with CKD and Decreased eGFR: A Multicenter Prospective Observational Study in Greece

SUPPLEMENTARY

SUPPLEMENTARY FIGURES

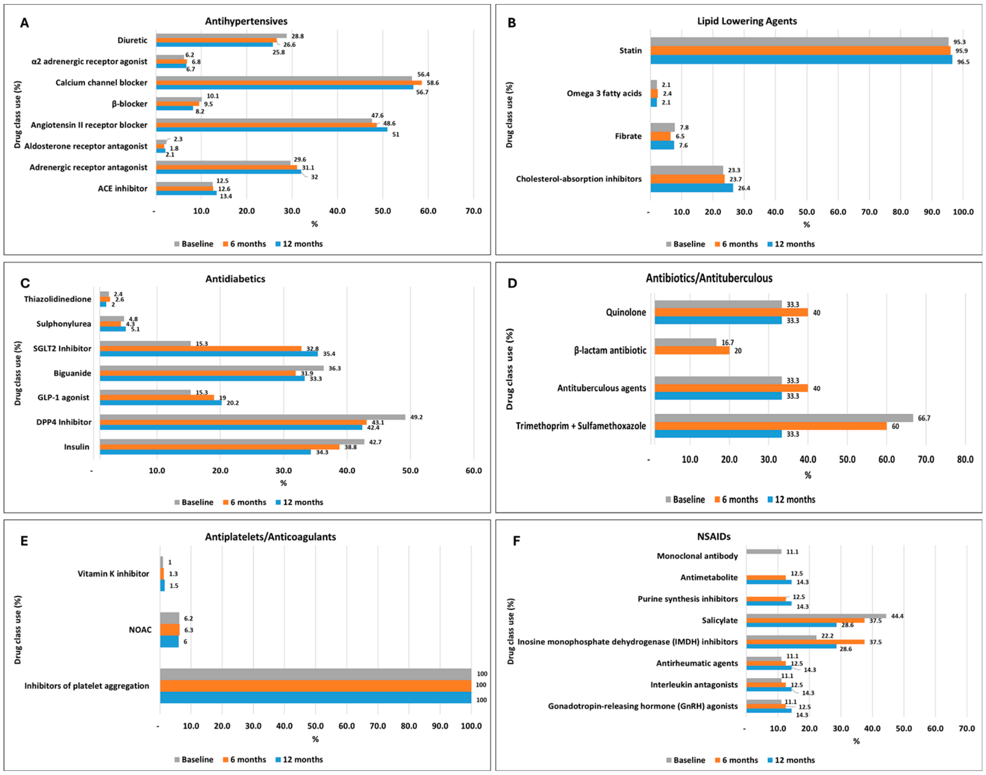

Figure S1. Drug subcategories administered across study visits for: A. Antihypertensives, B. Lipid lowering agents, C. Antidiabetics, D. Antiplatelets/Anticoagulants, E. Antibiotics/Antituberculous, F. NSAIDs. Percentages of patients receiving each treatment per study visit are presented.

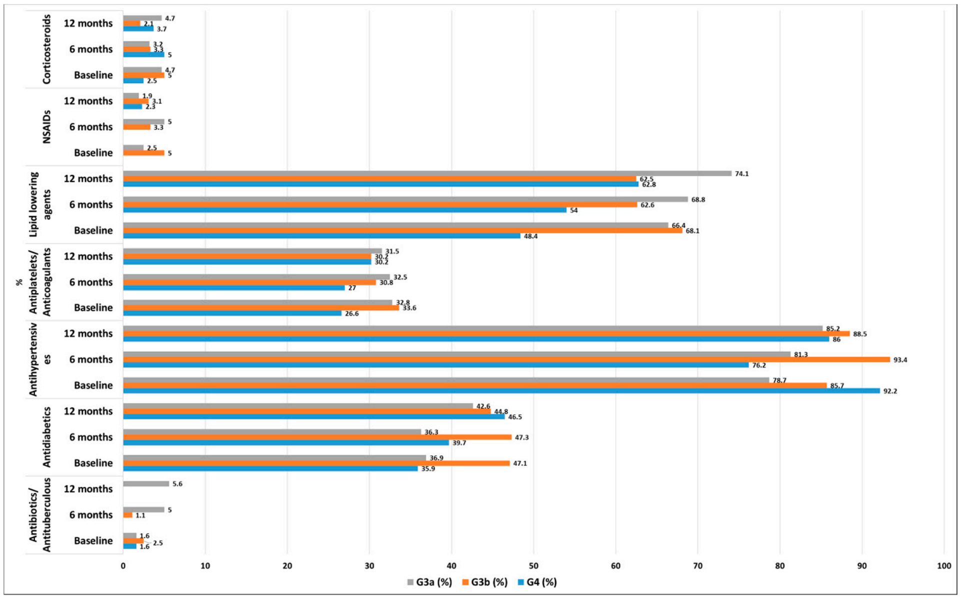

Figure S2. Patterns in overall use of A. Antihypertensives, B. Antidiabetics, C. Lipid Lowering agents, D. Antiplatelets/Anticoagulants, E. Antibiotics/Antituberculous, F. Corticosteroids, and G. NSAIDs, across CKD stages, stratified by study visit.

SUPPLEMENTARY TABLES

Table S1. Clinical monitoring of other laboratory parameters over time

	Baseline	6 months	12 months
Haematology [1]	N (%) = 256 (83.9%)	N (%) = 204 (77.3%)	N (%) = 228 (86.8%)
**Hemoglobin (g/dL)**			
N	256	204	195
Median (IQR)	12.8 (11.4, 14.1)	12.9 (11.6, 14.0)	12.6 (11.5, 13.8)
Missing, n (%)	22 (7.2%)	60 (22.7%)	30 (13.2%)
Not measured, n (%)	27 (8.9%)	-	-
**Hematocrit (%)**			
N	256	204	195
Median (IQR)	39.0 (35.0, 43.0)	40.0 (36.0, 44.0)	38.5 (34.5, 43.0)
Missing, n (%)	22 (7.2%)	60 (22.7%)	30 (13.2%)

	Baseline	6 months	12 months
Not measured, n (%)	27 (8.9%)	-	-
Biochemistry [1]	N (%) = 265 (86.9%)	N (%) = 211 (79.9%)	N (%) = 205 (89.9%)
**Serum albumin (g/dL)**			
N	216	176	172
Median (IQR)	4.2 (4.0, 4.5)	4.3 (4.1, 4.5)	4.3 (4.1, 4.5)
Missing, n (%)	4 (1.5%)	1 (0.5%)	2 (1.0%)
Not measured, n (%)	45 (17.0%)	34 (16.1%)	31 (15.1%)
**HbA1c (%)**			
N	117	106	98
Median (IQR)	6.1 (5.7, 6.9)	6.0 (5.7, 6.6)	6.1 (5.8, 6.8)
Missing, n (%)	10 (3.8%)	5 (2.4%)	4 (2.0%)
Not measured, n (%)	138 (52.1%)	100 (47.4%)	103 (50.2%)
**Total cholesterol (mg/dL)**			
N	171	145	141
Median (IQR)	169.0 (141.5, 194.5)	163.0 (139.0, 196.0)	165.0 (143.0, 193.0)
Missing, n (%)	7 (2.6%)	1 (0.5%)	-
Not measured, n (%)	87 (32.8%)	65 (30.8%)	64 (31.2%)
**HDL (mg/dL)**			
N	160	131	128
Median (IQR)	44.5 (35.0, 53.0)	47.0 (40.0, 57.5)	47.0 (40.0, 60.0)
Missing, n (%)	7 (2.6%)	1 (0.5%)	-
Not measured, n (%)	98 (37.0%)	79 (37.4%)	77 (37.6%)
**LDL (mg/dL)**			
N	163	141	146
Median (IQR)	88.0 (64.5, 116.5)	89.0 (65.0, 113.0)	89.0 (71.0, 107.0)
Missing, n (%)	7 (2.6%)	2 (0.9%)	-
Not measured, n (%)	95 (35.8%)	68 (32.2%)	59 (28.8%)

	Baseline	6 months	12 months
**Potassium (mEq/L)**			
N	258	210	202
Median (IQR)	4.6 (4.4, 5.0)	4.7 (4.3, 4.9)	4.7 (4.4, 5.0)
Missing, n (%)	-	-	-
Not measured, n (%)	7 (2.6%)	1 (0.5%)	3 (1.5%)
**Phosphorus (mg/dL)**			
N	229	192	178
Median (IQR)	3.5 (3.1, 4.0)	3.7 (3.2, 4.0)	3.8 (3.2, 4.1)
Missing, n (%)	3 (1.3%)	3 (1.4%)	1 (0.5%)
Not measured, n (%)	33 (12.5%)	16 (7.6%)	26 (12.7%)
**PTH (pg/dL)**			
N	157	111	128
Median (IQR)	69.0 (49.9, 117.0)	79.0 (55.0, 121.4)	71.2 (52.8, 125.6)
Missing, n (%)	10 (3.8%)	-	2 (1.0%)
Not measured, n (%)	98 (37.0%)	100 (47.4%)	75 (36.6%)
**Uric acid (mg/dL)**			
N	226	179	182
Median (IQR)	6.4 (5.4, 7.6)	6.3 (5.1, 7.2)	6.3 (5.3, 7.2)
Missing, n (%)	1 (0.4%)	2 (0.9%)	-
Not measured, n (%)	38 (0.1%)	30 (14.2%)	23 (11.2%)
**Urea (mg/dL)**			
N	260	206	202
Median (IQR)	65.0 (50.0, 90.0)	67.5 (52.0, 88.8)	64.0 (50.0, 90.0)
Missing, n (%)	-	1 (0.5%)	-
Not measured, n (%)	5 (1.9%)	4 (1.9%)	3 (1.5%)
Urinalysis: chemical characteristics [1]	N (%) = 224 (73.4%)	N (%) = 145 (54.9%)	N (%) = 228 (68.4%)
**Hematuria, n (%)**			
Positive	54 (24.1%)	41 (28.3%)	45 (28.8%)

	Baseline	6 months	12 months
Negative	167 (74.6%)	104 (71.7%)	111 (71.2%)
Not measured, n (%)	3 (1.3%)	-	-
**Pyuria, n (%)**			
Positive	62 (27.7%)	27 (18.6%)	34 (21.8%)
Negative	152 (67.9%)	117 (80.7%)	121 (77.6%)
Not measured, n (%)	10 (4.5%)	1 (0.7%)	1 (0.6%)
**Microorganisms**			
Positive	38 (17.0%)	14 (9.7%)	27 (17.3%)
Negative	143 (63.8%)	118 (81.4%)	111 (71.2%)
Missing, n (%)	3 (1.3%)		
Not measured, n (%)	40 (17.9%)	13 (9.0%)	18 (11.5%)
Urinalysis: analytical tests, n (%) [1]	132 (43.3%)	71 (26.9%)	56 (24.6%)
**Protein (mg/dL)**			
N	79	46	28
Median (IQR)	0 (0, 7.8)	2 (0, 82.5)	3.5 (0.15, 30)
Missing, n (%)	1 (0.8%)	-	-
Not measured, n (%)	52 (39.4%)	25 (35.2%)	28 (50.0%)
Vital signs [1]	N (%) = 262 (85.9%)	N (%) = 211 (79.9%)	N (%) = 200 (87.7%)
**Resting heart rate (bpm)**			
N	261	200	197
Median (IQR)	73.0 (67.0, 79.0)	72.0 (68.0, 78.0)	74.0 (69.0, 79.0)
Missing, n (%)	48 (15.7%)	12 (4.5%)	-
Not measured, n (%)	-	57 (21.6%)	-
**Systolic blood pressure (mmHg)**			
N	261	201	200
Median (IQR)	135.0 (123.0, 142.0)	132.0 (125.0, 142.0)	132.0 (125.0, 140.0)

	Baseline	6 months	12 months
Missing, n (%)	1 (0.4%)	10 (4.7%)	-
**Diastolic blood pressure (mmHg)**			
N	261	201	199
Median (IQR)	79.0 (70.0, 83.0)	79.0 (72.0, 82.0)	78.0 (70.0, 82.0)
Missing, n (%)	1 (0.4%)	10 (4.7%)	1 (0.5%)

[1] Calculations are based on the number of patients with available measurements for each test category.

Not measured n (%) refers to the number and percentage of values among patients for whom the corresponding test was intended and not assessed.

Missing n (%) refers to the number and percentage of missing values among patients for whom there was no info if they conducted the corresponding test.

bpm = beats per minute; HbA1c = glycated hemoglobin; HDL = high-density lipoprotein; IQR = Interquartile Range; LDL = low-density lipoprotein; PTH = parathyroid hormone; SD = standard deviation.

Table S2. Estimated changes from baseline in renal function and albuminuria over time based on linear mixed-effects models

	Baseline	6 months	Estimated difference at 6 months vs baseline (95% CI)	p-value	12 months	Estimated difference at 12 months vs baseline (95% CI)	p-value
Renal function	**N = 305**	**N = 264**			**N = 228**		
**Serum creatinine (mg/dL)**							
N	305	256			228		
Median (IQR)	1.7 (1.4, 2.1)	1.7 (1.4, 2.1)			1.7 (1.4, 2.1)		
Change vs baseline (LMM)			-1.2% (-3.6%, 1.4%)	0.4		2.9% (0.2%, 5.6%)	**0.04**
**CKD-EPI eGFR (ml/min/1.73 m²)**							
N	305	256			228		
Median (IQR)	40.0 (31.0, 50.0)	40.0 (30.0, 51.0)			40.0 (30.0, 50.0)		
Change vs baseline (LMM)			1.52 (0.3, 2.7)	**0.01**		0.2 (-1.1, 1.5)	0.8
**MDRD eGFR (ml/min/1.73 m²)**							
N	305	256			228		
Median (IQR)	37.9 (29.7, 46.6)	37.4 (28.9, 46.1)			37.6 (28.6, 46.0)		
Change vs baseline (LMM)			1.4 (0.3, 2.5)	**0.01**		0.15 (-1,0, 1.3)	0.8

	Baseline	6 months	Estimated difference at 6 months vs baseline (95% CI)	p-value	12 months	Estimated difference at 12 months vs baseline (95% CI)	p-value
Albuminuria	N = 305 [1]	N = 264			N = 228		
**Urinary albumin (mg/L)**							
N	305	142			157		
Median (IQR)	9.8 (2.0, 43.6)	13.5 (2.9, 40.8)			14.0 (2.5, 45.4)		
Change vs baseline (LMM)			-2.8% (-20.3%, 18.5%)	0.8		1.6% (-16.0%, 22.9%)	0.9
Missing, n (%)	0 (0.0%)	122 (46.2%)			71 (31.1%)		
**Urinary creatinine (g/dl)**							
N	302	145			158		
Median (IQR)	0.1 (0.05, 0.1)	0.1 (0.04, 0.09)			0.1 (0.05, 0.09)		
Change vs baseline (LMM)			-11.2% (-23.2%, 2.6%)	0.1		-2.6% (-15.3%, 12.1%)	0.7
Missing, n (%)	3 (1.0%)	119 (45.1%)			70 (30.7%)		
**UACR (mg/g)**							
N	302	142			155		
Median (IQR)	145.2 (28.9, 640.1)	253.4 (48.8, 638.8)			243.3 (43.1, 647.7)		

	Baseline	6 months	Estimated difference at 6 months vs baseline (95% CI)	p-value	12 months	Estimated difference at 12 months vs baseline (95% CI)	p-value
Change vs baseline (LMM)			12.2% (-12.3%, 43.5%)	0.4		10.6% (-12.8%, 40.2%)	0.4
Missing, n (%)	3 (1.0%)	122 (46.2%)			73 (32.0%)		

[1] Measured within 6 months from baseline.

CKD = chronic kidney disease; CKD-EPI = Chronic Kidney Disease Epidemiology Collaboration (equation for eGFR); eGFR = estimated glomerular filtration rate; IQR = Interquartile Range; MDRD = Modification of Diet in Renal Disease (equation for eGFR); SD = standard deviation; UACR = urinary albumin-to-creatinine ratio.

Table S3. Analysis on UACR change after SGLT2i administration

	Baseline	6 months	12 months
Patients receiving SGLT2i in total			
Albuminuria	N = 45	N = 41	N = 36
**Urinary albumin (mg/L)**			
N	45	24	28
Median (IQR)	2.7 (1.20, 15.0)	3.0 (1.1, 16.5)	2.5 (0.9, 14.0)
Missing, n (%)	-	17 (41.5%)	8 (22.2%)
**Urinary creatinine (g/dl)**			
N	45	24	28
Median (IQR)	0.06 (0.05, 0.09)	0.07 (0.06, 0.08)	0.06 (0.06, 0.09)
Missing, n (%)	-	17 (41.5%)	8 (22.2%)

**UACR (mg/g)**			
N	45	24	28
Median (IQR)	45.1 (20.3, 180.5)	52.4 (22.1, 240.6)	40.8 (18.9, 210.7)
Missing, n (%)	-	17 (41.5%)	8 (22.2%)

Non-diabetic patients receiving SGLT2i

Albuminuria	N = 15	N = 15	N = 12
**Urinary albumin (mg/L)**			
N	15	10	11
Median (IQR)	5.6 (2.6, 33.6)	18.0 (3.2, 49.0)	6.0 (2.4, 52.2)
Missing, n (%)	-	5 (33.3%)	1 (8.3%)
**Urinary creatinine (g/dl)**			
N	15	10	11
Median (IQR)	0.1 (0.05, 0.09)	0.1 (0.06, 0.07)	0.07 (0.06, 0.12)
Missing, n (%)	-	5 (33.3%)	1 (8.3%)
**UACR (mg/g)**			
N	15	10	11
Median (IQR)	70.3 (51.3, 466.4)	289.6 (67.3, 606.7)	47.4 (26.5, 553.5)
Missing, n (%)	-	5 (33.3%)	1 (8.3%)

Diabetic patients receiving SGLT2i

Albuminuria	N = 30	N = 26	N = 24
**Urinary albumin (mg/L)**			
N	30	14	17
Median (IQR)	2.2 (0.9, 8.3)	2.5 (0.9, 4.5)	2.0 (0.6, 6.7)
Missing, n (%)	-	12 (46.2%)	7 (29.2%)
**Urinary creatinine (g/dl)**			
N	30	14	17

Median (IQR)	0.06 (0.05, 0.09)	0.07 (0.06, 0.08)	0.06 (0.05, 0.08)
Missing, n (%)	-	12 (46.2%)	7 (29.2%)
**UACR (mg/g)**			
N	30	14	17
Median (IQR)	36.6 (15.9, 93.0)	35.6 (14.9, 84.0)	33.2 (13.6, 134.3)
Missing, n (%)	-	12 (46.2%)	7 (29.2%)

IQR = Interquartile Range; UACR = urinary albumin-to-creatinine ratio.

Table S4. Treatments Distribution During the Study Stratified by CKD Stage

	Stage G3a	Stage G3b	Stage G4
Baseline (Visit 1)	**N=122**	**N=119**	**N=64**
**Antidiabetic n (%)**	**N = 45 (36.9%)**	**N = 56 (47.1%)**	**N = 23 (35.9%)**
Insulin	22 (48.9%)	33 (58.9%)	18 (78.3%)
DPP4 Inhibitor	22 (48.9%)	33 (58.9%)	16 (69.6%)
GLP-1 agonist	8 (17.8%)	9 (16.1%)	2 (8.7%)
Biguanide	32 (71.1%)	18 (32.1%)	4 (17.4%)
SGLT2 Inhibitor	5 (11.1%)	13 (23.2%)	1 (4.3%)
Sulphonylurea	3 (6.7%)	2 (3.6%)	1 (4.3%)
Thiazolidinedione	3 (4.4%)	-	0 (0.0%)
**Antihypertensive n (%)**	**N = 96 (78.7%)**	**N = 102 (85.7%)**	**N = 59 (92.2%)**
ACE inhibitor	13 (13.5%)	16 (15.7%)	4 (6.8%)
Adrenergic receptor antagonist	22 (22.9%)	28 (27.5%)	26 (44.1%)
Aldosterone receptor antagonist	1 (1.0%)	3 (2.9%)	2 (3.4%)
Angiotensin II receptor blocker	64 (66.7%)	47 (46.1%)	15 (25.4%)
B-blocker	9 (9.4%)	12 (%)	11 (%)
Calcium channel blocker	46 (47.9%)	62 (60.8%)	42 (71.2%)

	Stage G3a	Stage G3b	Stage G4
Baseline (Visit 1)	**N=122**	**N=119**	**N=64**
α2 adrenergic receptor agonist	4 (4.2%)	4 (3.9%)	5 (8.5%)
Diuretic	27 (28.1%)	30 (29.4%)	20 (33.9%)
**Antiplatelet/Anticoagulant n (%)**	**N = 40 (32.8%)**	**N = 40 (33.6%)**	**N = 17 (26.6%)**
Inhibitors of platelet aggregation	38 (95.0%)	40 (100%)	17 (100.0%)
NOAC	5 (12.5%)	1 (2.5%)	-
Vitamin K inhibitor	-	-	1 (5.9%)
**Lipid Lowering Agent n (%)**	**N = 81 (66.4%)**	**N = 81 (68.1%)**	**N = 31 (48.4%)**
Cholesterol-absorption inhibitors	14 (17.3%)	14 (17.3%)	7 (22.6%)
Fibrate	7 (8.6%)	6 (7.4%)	2 (6.5%)
Statin	76 (93.8%)	78 (96.3%)	28 (90.3%)
Omega 3 fatty acids	2 (2.5%)	2 (2.5%)	0 (0.0%)
**NSAIDs n (%)**	**N = 3 (2.5%)**	**N = 6 (5.0%)**	**N=0 (0.0%)**
Gonadotropin-releasing hormone (GnRH) agonists	-	1 (16.7%)	-
Interleukin antagonists	-	1 (16.7%)	-
Antirheumatic agents	1 (33.3%)	-	-
Inosine monophosphate dehydrogenase (IMDH) inhibitors	1 (33.3%)	1 (16.7%)	-
Salicylate	2 (66.7%)	2 (33.3%)	-
Purine synthesis inhibitors	-	-	-
Antimetabolite	-	-	-
Monoclonal antibody	-	1 (16.7%)	-
**Costicosteroid n (%)**	**N = 3 (2.5%)**	**N = 6 (5.0%)**	**N = 3 (4.7%)**

Table S5. KDQOL-SF v1.3 Domain Scores Stratified by Visit and Comparative analysis (MMRM) between Study Visits

Subscale	N	Mean (SD)	Median	25th Percentile	75th Percentile	Change from baseline (p-value)	Change from 6 months (p-value)
Baseline (Visit 1)	304						
Burden of kidney disease	302	74.5 (23.5)	75.0	57.8	100.0	-	-
Cognitive function	302	85.0 (17.7)	93.3	80.0	100.0	-	-
Dialysis staff encouragement	3	100.0 (0.0)	100.0	100.0	100.0	-	-
Effects of kidney disease	301	85.2 (14.2)	87.5	78.1	96.9	-	-
Emotional well-being	304	68.8 (21.3)	72.0	52.0	85.0	-	-
Energy/fatigue	304	60.8 (23.2)	65.0	45.0	80.0	-	-
General health	304	53.0 (19.9)	55.0	40.0	70.0	-	-
Overall Health	300	68.9 (17.3)	70.0	60.0	80.0	-	-
Pain	304	74.2 (26.7)	77.5	55	100.0	-	-
Patient satisfaction	16	82.3 (12.9)	83.3	66.7	87.5	-	-
Physical functioning	304	65.4 (28.2)	70.0	45.0	90.0	-	-
Quality of social interaction	302	80.1 (18.6)	86.7	66.7	93.3	-	-
Role emotional	304	68.0 (42.5)	100.0	33.3	100.0	-	-
Role physical	302	63.2 (42.9)	100.0	25.0	100.0	-	-
SF-12 Mental Composite	297	48.3 (11.2)	51.6	41.2	57.8	-	-
SF-12 Physical Composite	297	44.2 (9.7)	45.3	36.9	52.9	-	-
Sexual function	75	81.0 (27.5)	100.0	62.5	100.0	-	-
Sleep	301	72.3 (18.2)	75.0	60.0	87.5	-	-

Subscale	N	Mean (SD)	Median	25th Percentile	75th Percentile	Change from baseline (p-value)	Change from 6 months (p-value)
Social function	304	72.0 (26.2)	75.0	50.0	100.0	-	-
Social support	300	85.1 (24.1)	100.0	83.3	100.0	-	-
Symptom/ problem list	302	85.3 (14.3)	89.1	77.3	95.4	-	-
6 months (Visit 2)	259						
Burden of kidney disease	259	75.3 (22.7)	81.2	62.5	93.8	0.753	-
Cognitive function	259	88.5 (15.0)	93.3	80.0	100.0	**0.003**	-
Dialysis staff encouragement	-	-	-	-	-	-	-
Effects of kidney disease	259	86.7 (13.4)	90.6	81.2	96.9	0.2	-
Emotional well-being	259	72.6 (20.1)	80.0	56.0	88.0	**0.005**	-
Energy/fatigue	259	64.3 (21.2)	70.0	50.0	80.0	**0.034**	-
General health	259	54.6 (20.1)	55.0	40.0	70.0	0.416	-
Overall Health	257	70.6 (16.7)	70.0	60.0	80.0	0.281	-
Pain	259	77.2 (23.7)	80.0	61.2	100.0	0.215	-
Patient satisfaction	5	66.7 (11.8)	66.7	66.7	66.7	0.232	-
Physical functioning	259	66.7 (28.5)	75.0	45.0	90.0	0.943	-
Quality of social interaction	259	81.4 (17.9)	86.7	66.7	93.3	0.565	-
Role emotional	259	73.4 (39.9)	100.0	33.3	100.0	0.135	-
Role physical	259	66.5 (42.6)	100.0	25.0	100.0	0.356	-
SF-12 Mental Composite	254	50.1 (10.0)	53.7	42.9	57.8	**0.024**	-
SF-12 Physical Composite	254	44.6 (9.9)	47.1	36.7	53.2	0.853	-

Subscale	N	Mean (SD)	Median	25th Percentile	75th Percentile	Change from baseline (p-value)	Change from 6 months (p-value)
Sexual function	72	84.9 (23.8)	100.0	75.0	100.0	0.359	-
Sleep	259	73.9 (17.5)	77.5	62.5	87.5	0.272	-
Social function	259	75.9 (25.1)	87.5	62.5	100.0	**0.02**	-
Social support	258	87.3 (22.8)	100.0	83.3	100.0	0.289	-
Symptom/ problem list	259	87.4 (13.0)	90.9	79.6	97.7	0.042	-
12 months (Visit 3)	222						
Burden of kidney disease	219	72.1 (24.4)	75.0	56.2	93.8	0.201	0.051
Cognitive function	218	85.8 (18.3)	93.3	80.0	100.0	0.889	**0.029**
Dialysis staff encouragement	-	-	-	-	-	-	-
Effects of kidney disease	217	83.4 (18.1)	90.6	78.1	96.9	0.15	**0.002**
Emotional well-being	220	70.9 (20.2)	76.0	56.0	88.0	0.283	0.335
Energy/fatigue	220	63.5 (21.5)	70.0	50.0	80.0	0.361	0.594
General health	220	54.8 (21.1)	55.0	45.0	70.0	0.502	0.997
Overall Health	212	71.4 (16.6)	80.0	60.0	80.0	0.072	0.725
Pain	220	76.6 (23.9)	80.0	57.5	100.0	0.413	0.95
Patient satisfaction	2	66.7 (0.0)	66.7	66.7	66.7	0.574	0.931
Physical functioning	220	65.9 (29.4)	70.0	48.8	90.0	0.899	0.743
Quality of social interaction	218	78.2 (21.0)	86.7	66.7	98.3	0.115	**0.011**
Role emotional	220	72.4 (40.0)	100.0	33.3	100.0	0.249	0.977
Role physical	218	65.7 (42.1)	100.0	25.0	100.0	0.595	0.948
SF-12 Mental Composite	211	50.3 (9.9)	53.0	43.9	57.9	0.077	0.956

Subscale	N	Mean (SD)	Median	25th Percentile	75th Percentile	Change from baseline (p-value)	Change from 6 months (p-value)
SF-12 Physical Composite	211	44.5 (10.0)	47.0	36.1	53.0	0.994	0.824
Sexual function	62	83.3 (24.2)	100.0	75.0	100.0	0.716	0.867
Sleep	217	73.7 (17.8)	77.5	62.5	87.5	0.238	0.984
Social function	220	75.7 (25.7)	87.5	62.5	100.0	0.121	0.842
Social support	214	85.3 (24.4)	100.0	66.7	100.0	0.947	0.53
Symptom/ problem list	219	86.6 (14.2)	90.9	79.6	95.4	0.444	0.55

Table S6. EQ-5D-5L Metrics during the Study, and Comparative Analysis between Study Visits

EQ-5D-5L Metrics	N	Mean (SD)	Median	25th Percentile	75th Percentile	Change from baseline (p-value)	Change from 6 months (p-value)
Baseline (Visit 1)	304						
EQ-5D-5L Index Value	301	0.8 (0.2)	0.9	0.8	1.0	-	-
EQ-5D-5L VAS Score	304	71.9 (17.9)	75.0	60.0	85.0	-	-
6 months (Visit 2)	261						
EQ-5D-5L Index Value	259	0.8 (0.2)	0.9	0.8	1.0	0.596	-
EQ-5D-5L VAS Score	261	74.1 (16.8)	75.0	65.0	85.0	0.085	-
12 months (Visit 3)	226						
EQ-5D-5L Index Value	224	0.8 (0.2)	0.9	0.8	1.0	0.857	0.327
EQ-5D-5L VAS Score	226	74.8 (15.8)	80.0	65.0	85.0	0.019	0.772
